# *Cymbopogon citratus* and *Camellia sinensis* extracts selectively induce apoptosis in cancer cells and reduce growth of lymphoma xenografts *in vivo*

**DOI:** 10.18632/oncotarget.22502

**Published:** 2017-11-18

**Authors:** Cory Philion, Dennis Ma, Ivan Ruvinov, Fadi Mansour, Christopher Pignanelli, Megan Noel, Ammar Saleem, John Arnason, Mark Rodrigues, Inderpal Singh, Jesse Ropat, Siyaram Pandey

**Affiliations:** ^1^ Department of Chemistry and Biochemistry, University of Windsor, Windsor, Ontario N9B 3P4, Canada; ^2^ Department of Biology, University of Ottawa, Ottawa, Ontario K1N 6N5, Canada

**Keywords:** cancer, lymphoma, leukemia, nutraceuticals, oxidative stress

## Abstract

Cancer cells are reported to have elevated levels of reactive oxygen species (ROS) and are highly dependent on cellular defense mechanisms against oxidative stress. Numerous nutraceuticals and natural polyphenolic compounds have a wide range of abilities to alter cellular redox states with potential implications in various diseases. Furthermore, therapeutic options for cancers are mostly nonselective treatments including genotoxic or tubulin-targeting compounds. Some of the natural extracts, containing multiple bioactive compounds, could target multiple pathways in cancer cells to selectively induce cell death. *Cymbopogon citratus* (lemongrass) and *Camellia sinensis* (white tea) extracts have been shown to have medicinal properties, however, their activity against lymphoma and leukemia, as well as mechanistic details, have not been fully characterized. Herein, we report potent anti-cancer properties in dose and time-dependent manners of ethanolic lemongrass and hot water white tea extracts in lymphoma and leukemia models. Both extracts were able to effectively induce apoptosis selectively in these human cancer cell types. Interestingly, ethanolic lemongrass extract induces apoptosis primarily by the extrinsic pathway and was found to be dependent on the generation of ROS. Conversely, apoptotic induction by hot water white tea extract was independent of ROS. Furthermore, both of these extracts caused mitochondrial depolarization and decreased rates of oxygen consumption in lymphoma and leukemia cells, leading to cell death. Most importantly, both these extracts were effective in reducing tumor growth in human lymphoma xenograft models when administered orally. Thus, these natural extracts could have potential for being nontoxic alternatives for the treatment of cancer.

## INTRODUCTION

Conventional chemotherapeutics for the treatment of cancer lead to serious side effects due to the nonselective nature of these treatments, targeting features common to both healthy and cancerous cells. Patients undergoing chemotherapeutic regimes experience difficulties coping with the toxicity of their treatments, and cancers may develop resistance to treatment, limiting their therapeutic potential. The oxidative and mitochondrial vulnerabilities unique to cancer cells, such as their increased dependence on aerobic glycolysis (Warburg effect), can be exploited. New approaches with selective therapies that circumvent the side effects of conventional treatments can be developed, targeting the aforementioned vulnerabilities. Cancerous cells may use a differential pathway of metabolism; indeed, it has been reported that they generate higher amounts of reactive oxygen species (ROS). Furthermore, they have unregulated anti-oxidative defense mechanisms, and an increase in the production of ROS can trigger apoptosis [1, 2, 3]. Moreover, cancerous mitochondria have been shown to be selectively susceptible to certain compounds, further indicating that there are vulnerabilities unique to cancer cells [4, 5].

There are several processes that result in the formation of reactive oxygen species (ROS), as they are natural products of many cellular metabolic reactions. One such reaction is through oxidative phosphorylation during normal cell respiration. During normal cell respiration, where electrons are transferred down the electron transport chain (ETC), an improper reduction reaction of oxygen can occur such that a superoxide radical is formed instead of water. Usually this rare occurrence is corrected by cellular scavenger systems, however, when cell scavenger systems are overwhelmed, ROS levels become increasingly high. This imbalance of ROS and radical scavenging mechanisms is referred to as oxidative stress and can lead to severe damage and, in extreme cases, apoptosis [1, 2].

There are two major pathways of apoptosis, termed the intrinsic and extrinsic pathways [4]. The intrinsic pathway is usually activated by some sort of intracellular stress. This causes the mitochondrial membrane pores to open, inducing mitochondrial membrane potential dissipation and the release of apoptogenic factors like cytochrome c [4, 6, 7]. Cytochrome c release leads to the activation of caspase-9, which in turn activates caspase-3 and yields the degradation of internal cellular structures and apoptosis execution. By contrast, the extrinsic pathway is initiated through the interaction of a death receptor and its associated ligand. There are several death receptors as their associated ligands, such as TNFR1/TNF-alpha and Fas/FasL. These receptors can initiate intracellular, pro-apoptotic signalling cascades, which can activate caspase-8 and ultimately caspase-3 [4, 6, 7].

Natural extracts are isolated from various food sources and plants, and are utilized for medicinal properties [8]. *Cymbopogon citratus*, commonly referred to as lemongrass, is known to have several biologically active compounds, such as citral, which is thought to have anti-mutagenic, anti-proliferative, and anti-parasitic properties [9]. Additionally, free radical scavenging is also associated with this nutraceutical [9]. As of yet, the anti-cancer properties of lemongrass have not been fully characterized.

White tea is derived from the immature leaves of the *Camellia sinensis* plant species. It is known to contain a distinct group of polyphenols specifically categorized as epicatechins, which are thought to be the main contributors to the health benefits attributed to white tea [10]. The four major epicatechins found in white tea are epicatechin, epicatechin-3-gallate, epigallocatechin, and epigallocatechin-3-gallate [10]. It is thought that these bioactive catechins are able to interact with ROS to quench them *in vitro* [11]. As ROS have been linked to several progressive disease states, it is thought that the epicatechins in white tea can be used as a possible treatment. Currently, the anti-cancer and free radical scavenging properties of these compounds are being evaluated [10, 12].

In this work, lemongrass and white tea extracts were investigated for their potential anti-cancer activity in human lymphoma and leukemia models. Both extracts were able to reduce viability and selectively induce apoptosis in lymphoma and leukemia cells *in vitro*. Interestingly, both extracts were able to induce the extrinsic pathway of apoptosis in lymphoma and leukemia cells, but apoptotic induction by lemongrass extract was dependent on the generation of ROS while white tea extract was independent of oxygen radical production. Moreover, both extracts were effective in reducing growth of lymphoma xenographs, caused death of tumor cells, and reduced lymphoma cell proliferation *in vivo*. These findings present potentially safe and potent anti-cancer natural extracts with one (lemongrass extract) exploiting oxidative vulnerabilities of cancer cells to selectively induce apoptosis in malignant cells.

## RESULTS

### Lemongrass and white tea extracts reduce viability of lymphoma cells

**Figure 1:**
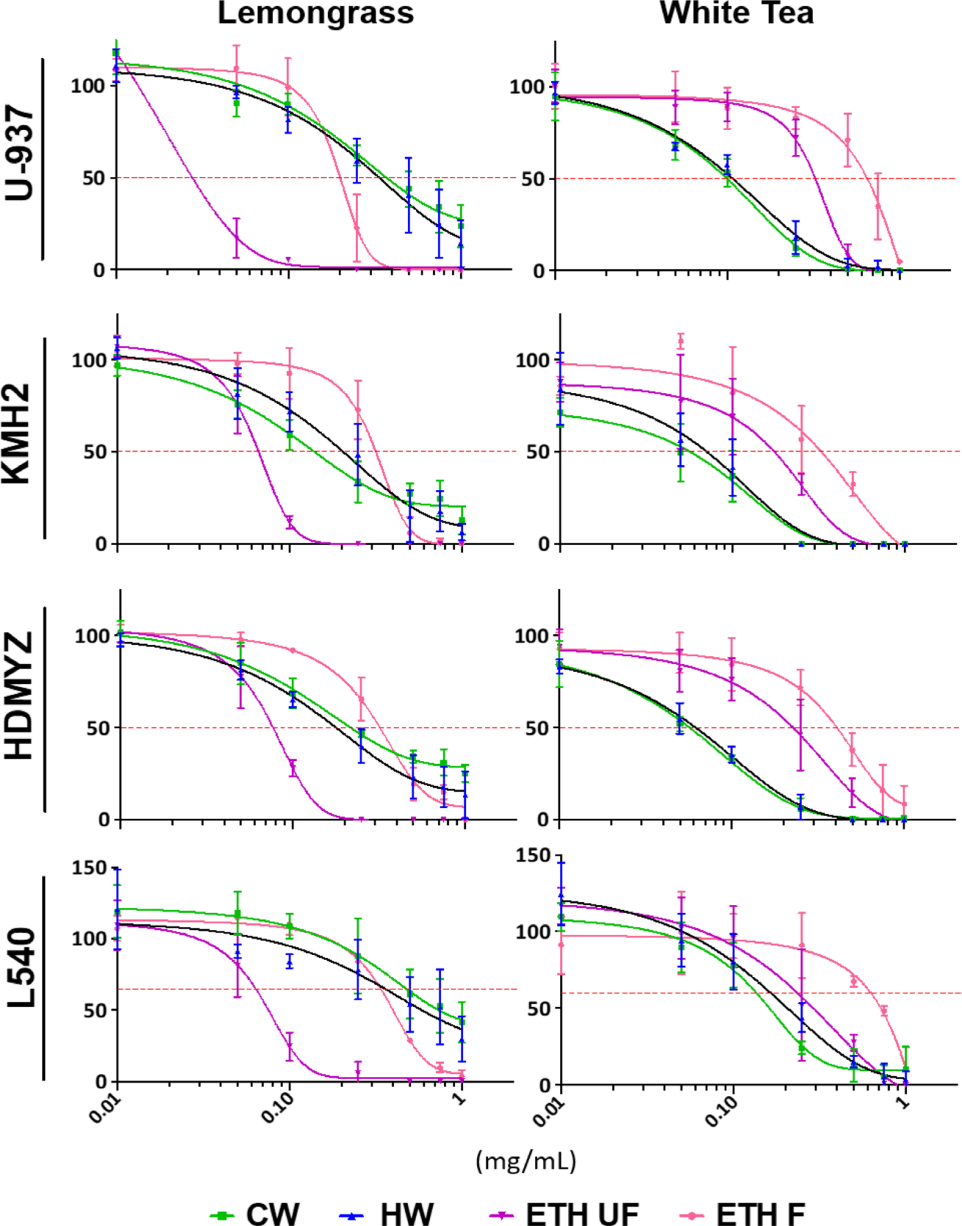
Lemongrass and white tea extracts display broad efficacy in reducing cell viability in 4 lymphoma cell lines Cells were treated with various doses of lemongrass and white tea extracts prepared with cold water, hot water, unfiltered ethanol, and filtered ethanol for 48 hours. Following treatment, WST-1 cell proliferation assay was used and the absorbance at 450 nm was measured. Y-axis is the percent mean ± SD from three independent experiments to the control and the x-axis is the concentrations used in milligram/milliliter. Results graphed using the log(inhibitor) vs. response - Variable slope (four parameters) curve using GraphPrism6.

Cold water (CW), hot water (HW), ethanolic-filtered (ETH-F), and ethanolic-unfiltered (ETH-UF) extracts of lemongrass and white tea were prepared as described in the materials and methods. To evaluate the cytotoxicity of lemongrass and white tea extracts on various Hodgkin and non-Hodgkin’s lymphoma cell lines, the water-soluble tetrazolium-1 (WST-1) assay was utilized. Both lemongrass and white tea extracts reduced the viability of all the tested lymphoma cell lines in a dose-dependent manner (Figure 1). In particular, the ETH-UF lemongrass and HW white tea extracts were the most effective. EC_50_ values for the different extracts and cell lines are available in Table 1. Interestingly, the non-Hodgkin’s lymphoma cell line, U-937, was sensitive to ETH-UF lemongrass extract at concentrations below 0.05 mg/mL whereas the Hodgkin’s lymphoma cell lines, KMH2, HDMYZ, and L540, were sensitive to ETH-UF lemongrass extract at concentrations below 0.1 mg/mL. Both the non-Hodgkin’s and Hodgkin’s lymphoma cell lines displayed similar sensitivity to HW and CW white tea extracts. Thus, ETH-UF lemongrass extract and HW white tea extract were the main focus for further evaluation and characterization for this study.

**Table 1:** EC_50_ values as calculated by WST-1 cell proliferation assay

Cell Line	EC_50_ Values (mg/mL)							
	Lemongrass				White Tea			
	CW	HW	EtOH UF	EtOH F	CW	HW	EtOH UF	EtOH F
	0.445231	0.390165	0.026015	0.188068	0.092383	0.101777	0.30335	0.638006
**U-937**	±	±	±	±	±	±	±	±
	0.150475	0.195853	0.010113	0.05305	0.015835	0.006124	0.016528	0.116234
	0.143711	0.211937	0.063408	0.298615	0.056853	0.066717	0.151296	0.280231
**KMH2**	±	±	±	±	±	±	±	±
	0.059122	0.099757	0.008652	0.049715	0.027818	0.025221	0.070172	0.114647
	0.212369	0.176398	0.072506	0.31694	0.054756	0.059655	0.213992	0.397054
**L540**	±	±	±	±	±	±	±	±
	0.034857	0.030158	0.012758	0.064166	0.00479	0.00874	0.069336	0.016837
	0.628508	0.500629	0.071518	0.383979	0.133725	0.197496	0.236157	0.695556
**HDMYZ**	±	±	±	±	±	±	±	±
	0.45653	0.281738	0.015451	0.001378	0.016334	0.000204	0.146421	0.011178

Four different lymphoma cell lines were treated for 48 hours with different extracts at several doses as described in the materials and methods. Cell viability was measured using the WST-1 assay as described in the legend of Figure 1 and EC_50_ values were calculated using GraphPrism6 and Microsoft Excel.

### Lemongrass and white tea extracts induce apoptosis in hodgkin and non-Hodgkin’s lymphoma cells

**Figure 2:**
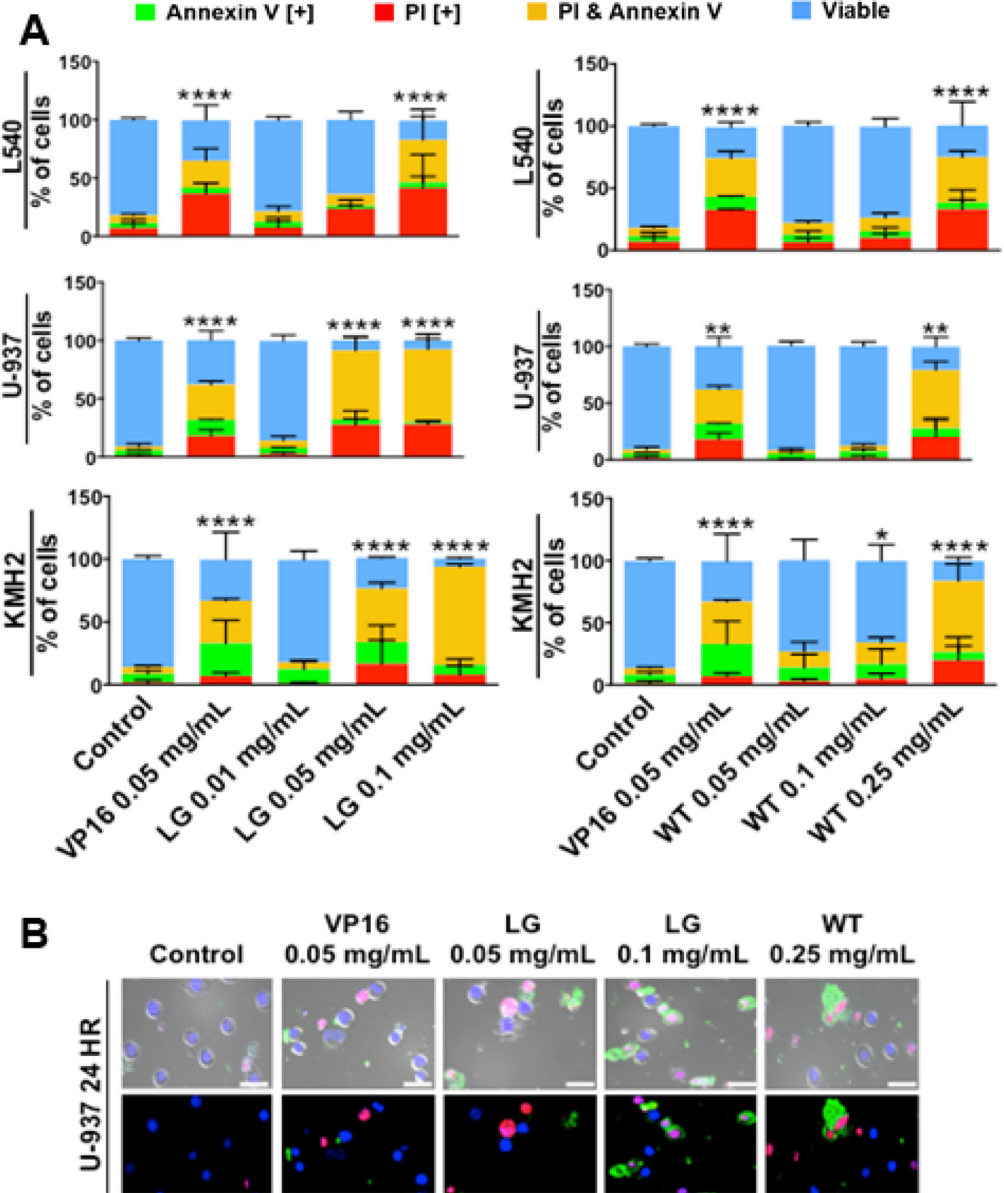
Lemongrass and white tea extracts induce apoptosis in several lymphoma cell lines; following treatment with specified doses, cells were stained for annexin V and PI (**A**) Lymphoma cell lines tested at 48 hours. Results were obtained using image-based cytometry with the Y-axis representative of percent of cells positive for Annexin V (green), PI (red), Annexin V and PI (yellow), or negative for both Annexin V and PI (blue). Values are expressed as a mean ± SD from three independent experiments. (**B**) U-937 micrographs at 24 hours. Top: Bright field and fluorescent merged images at 400x magnification. Bottom: Fluorescent images stained with Annexin V (green), PI (red), and Hoechst (blue) at 400× magnification. Scale bar is 50 microns. Images are representative of three independent experiments. Values are expressed as a mean ± SD from three independent experiments. Statistical calculations were performed using Two-Way ANOVA multiple comparison. ^*^p < 0.05 vs. Control, ^**^p < 0.01 vs. Control, ^****^p < 0.0001 vs. Control.

**Figure 3:**
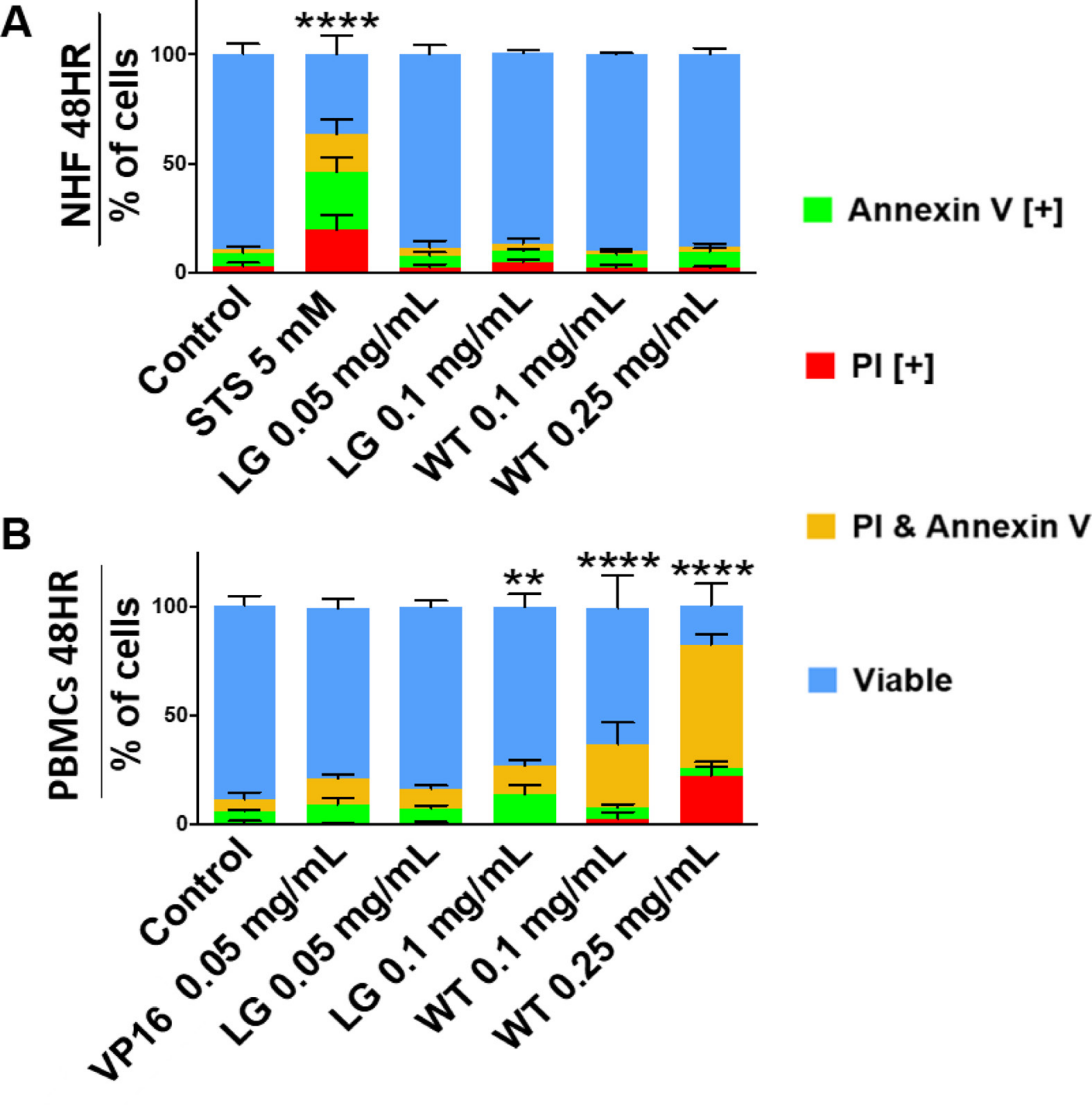
Lemongrass and white tea extracts do not induce apoptosis in non-cancerous cells (**A**) Normal human skin fibroblasts and (**B**) peripheral blood nuclear cells (from healthy individuals) were tested at 48 hours. Following treatment with specified doses, cells were stained for Annexin V and PI. Results were obtained using image-based cytometry with the Y-axis representative of percent of cells positive for Annexin V (green), PI (red), Annexin V and PI (yellow), or negative for both Annexin V and PI (blue). Values are expressed as a mean ± SD from three independent experiments. Statistical calculations were performed using Two-Way ANOVA multiple comparison. ^****^p < 0.0001 vs. Control.

To assess the ability of lemongrass and white tea extracts to induce apoptosis in lymphoma cells, cells were stained with Annexin V and propidium iodide, general markers of apoptosis, and subjected to image-based cytometry and microscopy following treatment with the extracts. Following treatment for 48 hours, both lemongrass and white tea extracts were effective in inducing apoptosis in lymphoma cells (Figure 2A).Notably, lemongrass extract was able to cause significant induction of apoptosis at a level (0.05 mg/mL) comparable to the standard chemotherapeutic VP16 in non-Hodgkin’s U-937 and Hodgkin’s KMH2 lymphoma cell lines. Fluorescent microscopy following treatment with lemongrass and white tea extracts and VP16 for 24 hours also revealed these cell death markers, along with apoptotic morphology in U-937 lymphoma cells, including cell shrinkage, membrane blebbing, and nuclear condensation (Figure 2B). Most importantly, there was minimal to no observable apoptotic induction with normal human fibroblast cells at doses of lemongrass and white tea extracts that were highly cytotoxic to lymphoma cells after 48 hours comapred to the positive control staurosporin (STS) (Figure 3A). Furthermore, in order to have a similar counterpart to lymphoma cells, we used peripheral nucleated blood cells (PNBCs) obtained from healthy individuals as a noncancerous control. These results further indicated that lemongrass extract showed minimal apoptotic effect on these cells, however white tea extract did induce apoptosis at higher doses (Figure 3B). Therefore, lemongrass showed excellent selectivity to cancer cells whereas white tea extract was toxic to PNBCs at higher doses.

### Lemongrass and white tea extracts cause mitochondrial depolarization and decreased rates of oxygen consumption in lymphoma cells

**Figure 4:**
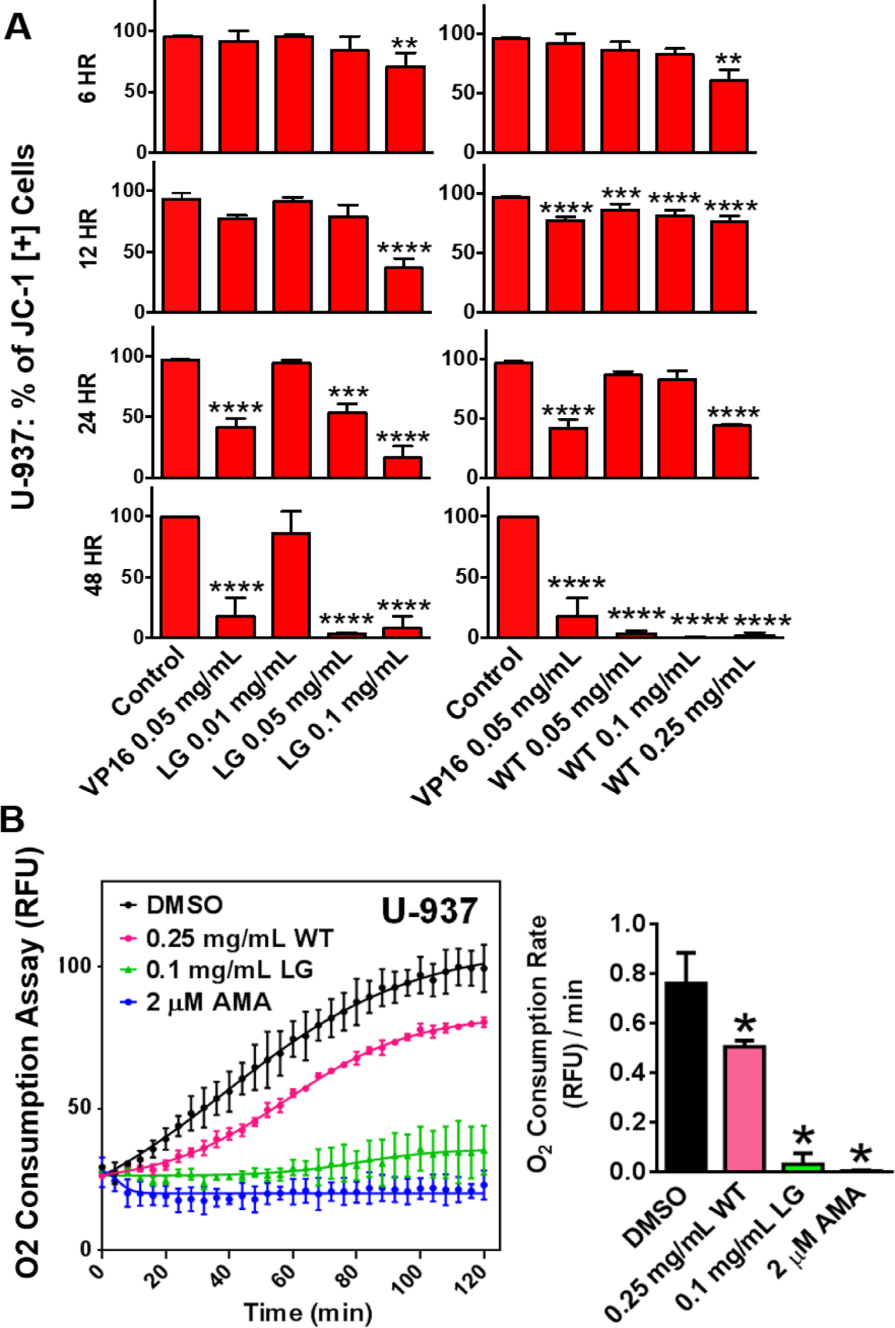
Lemongrass and white tea extracts cause mitochondrial depolarization and decreased rates of oxygen consumption in lymphoma cells (**A**) Lymphoma cells were plated and allowed to incubate overnight. Following overnight incubation, cells were treated for 6, 12, 24, and 48 hours. To monitor mitochondria potential cells were incubated with JC-1 for 30 minutes before analysis. Results were obtained using image-based cytometry with the Y-axis representative of percent of cells positive for JC-1 expressed as a mean ± SD from three independent experiments. (**B**) The MitoXpress^®^ Xtra - Oxygen Consumption Assay was used to monitor oxygen consumption via fluorescence generation as an indicator of mitochondrial function. U-937 lymphoma cells were treated with white tea extract (WT), lemongrass extract (LG), and antimycin A (AMA), and the fluorescent MitoXpress^®^ reagent was added monitored at Ex. 380 nm and Em. 650, every 2 minutes for 2 hours at 37°C. Oxygen consumption rates were calculated by measuring the slopes of the linear regions of the oxygen consumption curves. Values are expressed as mean ± SD from at least 3 independent experiments. ^*^p < 0.05 vs. Control, ^**^p < 0.01 vs. Control, ^***^p < 0.001 vs. Control, ^****^p < 0.0001 vs. Control.

Mitochondria play a key role in apoptosis, which can be triggered by mitochondrial dysfunction. This can lead to the permeabilization of the mitochondrial membrane, the release of apoptogenic factors, and the induction of apoptosis [13]. To monitor mitochondrial stability and depolarization, the fluorescent JC-1 assay was used. At time points as early as six and 12 hours, lemongrass and white extracts were able to decrease the percentage of cells positive for the JC-1 dye, and increasingly drastic reductions were observed at the 24 and 48 hour time-point (Figure 4A). This result indicates the collapse of mitochondrial potential in cells treated with lemongrass and white tea extracts.

To further investigate mitochondrial function directly, following treatment with lemongrass and white tea extracts, oxygen consumption was evaluated as described in materials and methods. Lemongrass and white tea extracts significantly decreased the rate of oxygen consumption in non-Hodgkin’s U-937 lymphoma cells (Figure 4B). Antimycin A (AMA) inhibits complex III of the ETC, and so it was used as a positive control in evaluating the disruption of oxygen consumption. This finding indicates that lemongrass and white tea extracts effectively decrease oxygen consumption and, therefore, mitochondrial function.

### Lemongrass extract is dependent on the production of oxidative stress to induce apoptosis

**Figure 5:**
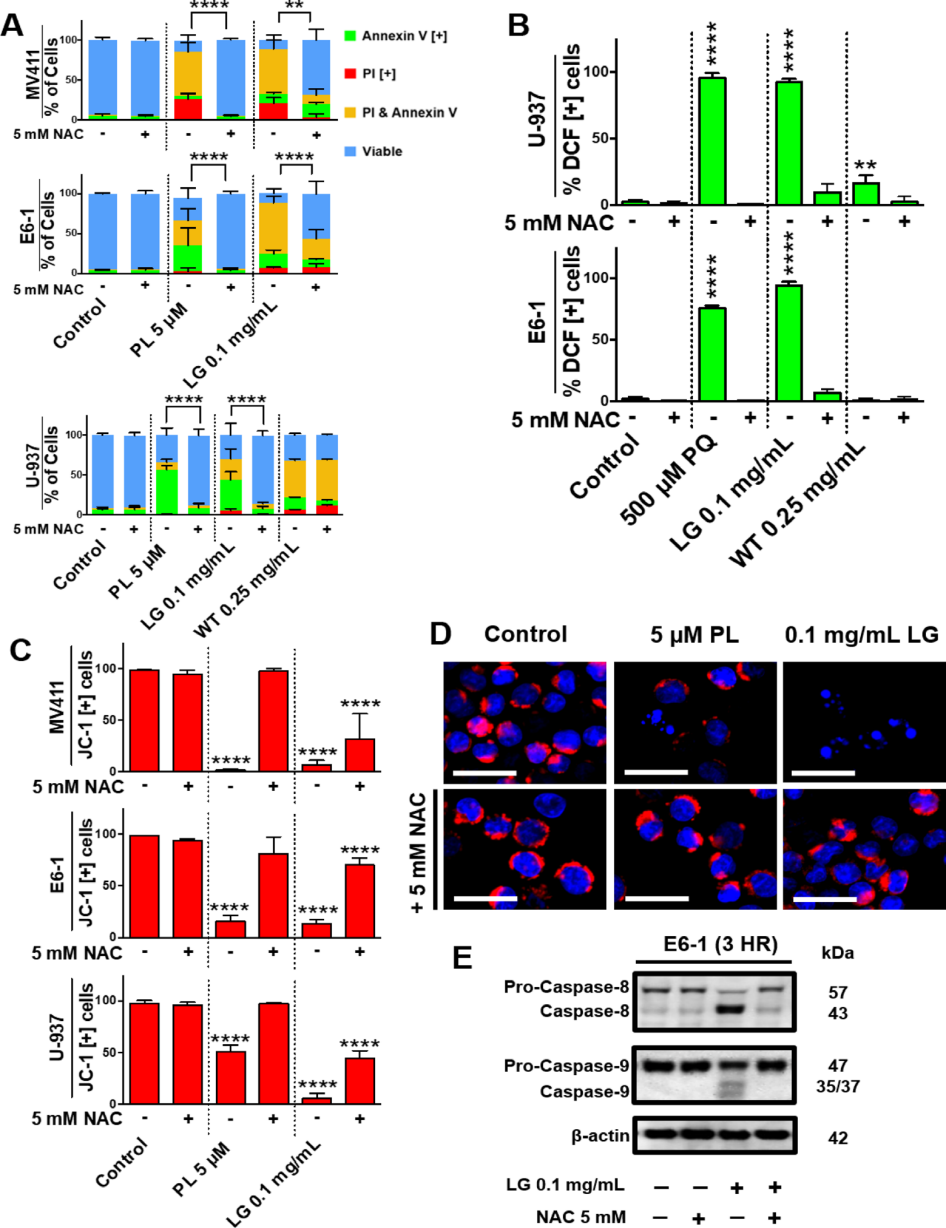
Lemongrass extract is dependent on the production of oxidative stress to induce apoptosis (**A**) MV-4-11, E6-1, and U-937 cells were treated with piperlongumine (PL), LG, or WT with or without the antioxidant NAC for 48 hours. Following treatment, cells were stained for Annexin V and PI. Results were obtained using image-based cytometry with the Y-axis representative of percent of cells positive for Annexin V (green), PI (red), Annexin V and PI (yellow), or negative for both Annexin V and PI (blue). Values are expressed as a mean ± SD from three independent experiments. (**B**) U-937 and E6-1 cells were treated with H2DCFDA following treatments with paraquat (PQ), LG, or WT with or without the antioxidant NAC for 3 hours. Results were obtained using the image-based cytometry with the Y-axis representative of percent of cells positive for DCF. Values are expressed as a mean ± SD from three independent experiments. (**C**) MV-4-11, E6-1, and U-937 cells were plated and allowed to incubate overnight. Following overnight incubation, cells were treated for 48 hours with or without NAC. To monitor mitochondria potential cells were incubated with JC-1 for 30 minutes before analysis. Results were obtained using image-based cytometry with the Y-axis representative of percent of cells positive for JC-1 expressed as a mean ± SD from three independent experiments. (**D**) U-937 micrographs at 48 hours. Top: Fluorescent images of cells without NAC stained with JC-1 (red) and Hoechst (blue) at 400x magnification. Bottom: Fluorescent images of cells with NAC stained with JC-1 (red) and Hoechst (blue) at 400x magnification. Scale bar is 25 microns. Images are representative of three independent experiments. (**E**) E6-1 cells were treated for 3 hours with LG with or without NAC, lysed, and subjected to SDS-PAGE. Cells were then transferred to a PVDF membrane and probed for the specific proteins. Bands were visualized with a chemiluminescence reagent. Statistical calculations were performed using Two-Way ANOVA multiple comparison for (A) and One-Way ANOVA multiple comparison for (B–C). ^**^p < 0.01 vs. Control, ^****^p < 0.0001 vs. Control.

Further investigating the mechanism of induction of apoptosis by these extracts, we observed that lemongrass extract induced production of ROS in several blood cancer cell lines, such as non-Hodgkin’s lymphoma U-937 cells and E6-1 Jurkat cells, as indicated by an increase in percent of cells positive for DCF, similar to the positive control piperlongumine (PL) (Figure 5A). In contrast, white tea extract showed minimal to no increase of ROS production in these cell lines (Figure 5A). We wanted to investigate if this increase in oxidative stress is essential in the downstream effects of lemongrass extract for the induction of apoptosis. When pre-treated with the potent antioxidant N-acetyl cysteine (NAC), there was a near complete inhibition of markers for apoptosis and mitochondrial destabilization across numerous cancerous cell lines following treatment with lemongrass extract, similar to the positive control paraquat (PQ). The markers for apoptosis were however unaffected following treatment with white tea extract (Figure 5B). Further experimentation with lemongrass extract investigated the role of oxidative stress in mitochondrial destabilization after treatment of three blood cancer cell lines (MV-4-11, E6-1, U-937) with lemongrass extracts and NAC, as indicated by a decrease in percent cells positive for JC-1 relative to the control (Figure 5C). When pre-treated with NAC, leukemia cells showed stabilized mitochondrial membrane potential in a qualitative microscopy study as indicated by the presence of red JC-1 dye around the blue Hoechst-stained DNA **(**Figure 5D). Furthermore, these results are complemented by the reduction in cleavage of caspase-8 and -9 by NAC co-treatment (Figure 5E). These results indicate that lemongrass and white extracts induce cell death by different mechanistic pathways; lemongrass extract appears to be dependent on oxidative stress for the induction of apoptosis whereas white tea extract is independent of oxidative stress.

### Functioning FADD Protein is required to induce apoptosis in cancer cells treated with lemongrass extract

**Figure 6:**
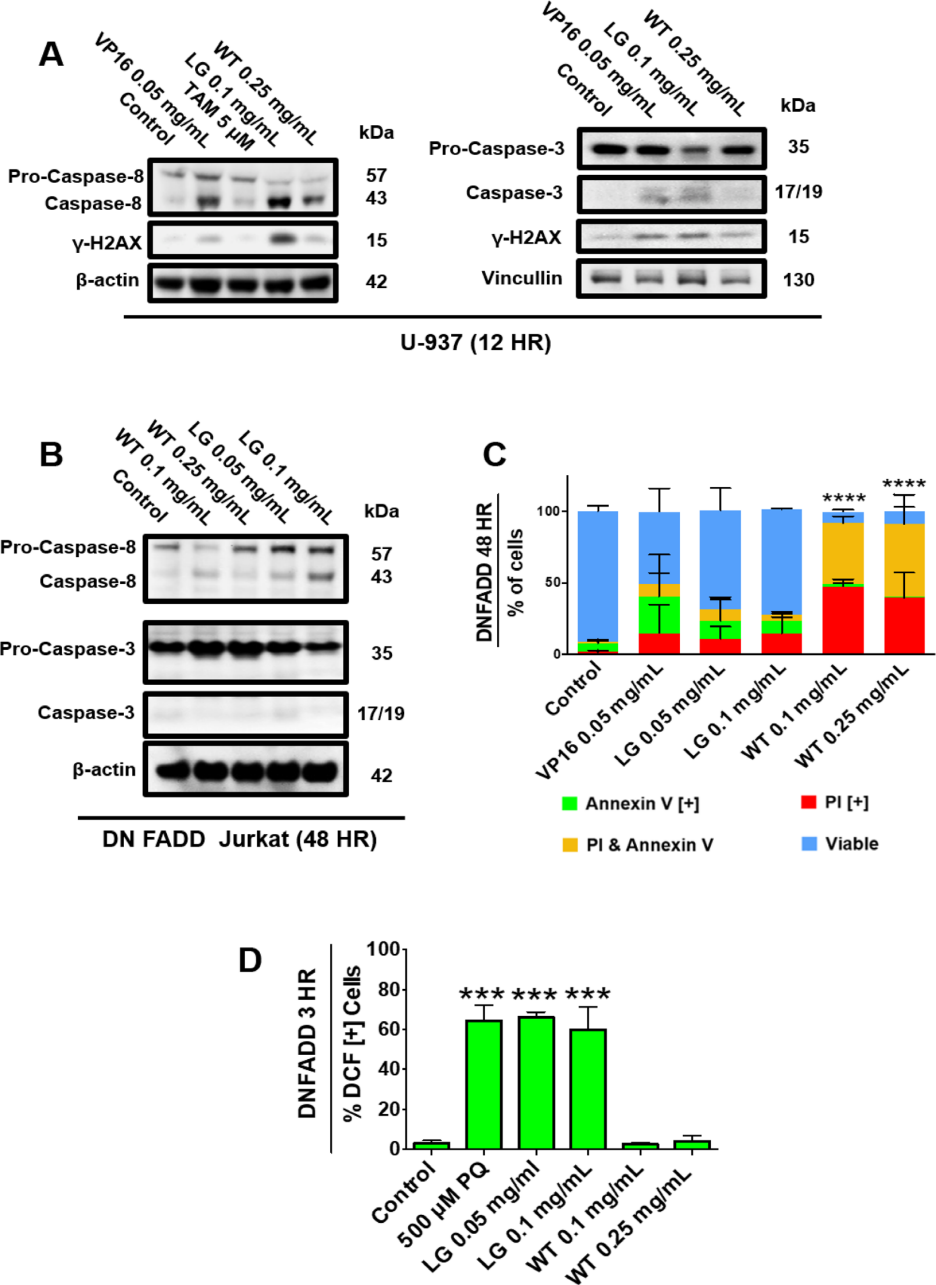
Functioning FADD protein is required to induce apoptosis in cancer cells treated with lemongrass extract (**A**) U-937 and (**B**) DN FADD Jurkat cells were treated for 12 hours and 48 hours, respectively, with the specified treatments, lysed, and subjected to SDS-PAGE. Cells were then transferred to a PVDF membrane and probed for the specific proteins. Bands were visualized with a chemiluminescence reagent. (**C**) DN FADD Jurkat cells were treated for 48 hours with the specified doses and stained with Annexin V and PI. Results were obtained using image-based cytometry with the Y-axis representative of percent of cells positive for Annexin V (green), PI (red), Annexin V and PI (yellow), or negative for both Annexin V and PI (blue). Values are expressed as a mean ± SD from three independent experiments. (**D**) DN FADD Jurkat cells were treated with H2DCFDA following treatments with paraquat (PQ), LG, or WT for 3 hours. Results were obtained using the image-based cytometry with the Y-axis representative of percent of cells positive for DCF. Values are expressed as a mean ± SD from three independent experiments. Statistical calculations were performed using Two-Way ANOVA multiple comparison for (C) and One-Way ANOVA multiple comparison for (D). ^***^p < 0.001 vs. Control, ^****^p < 0.0001 vs. Control.

In order to investigate the role of specific death pathways, we probed for caspase activation in non-Hodgkin’s U-937 lymphoma cells and E6-1 Jurkat cells lacking a functional Fas-associated protein with Death Domain (dnFADD Jurkat). Western blot analysis indicated that treatment with lemongrass extract activated caspase-8 in both cell lines, but caspase-3 was only activated in U-937 cells (Figure 6A, 6B). As a result, cell death by lemongrass extract was inhibited in the dnFADD cells (Figure 6C). Contrastingly, white tea extract maintained its ability to induce apoptosis in the dnFADD cell line and without caspase-3 activation (Figure 6B, 6C). Interestingly, lemongrass extract maintained its ability to produce ROS and white tea extract continued to show no increase in ROS production in the dnFADD cells (Figure 6D). These results indicate that lemongrass extract requires a functioning FADD protein for its apoptotic activity whereas white tea extract exhibits apoptotic activity independent of the FADD protein, and further corroborate the hypothesis that lemongrass and white tea extract induce cell death by different mechanistic pathways.

### Orally administered lemongrass and white tea extract reduce tumor size in lymphoma xenograft model in immunocompromised mice

**Figure 7:**
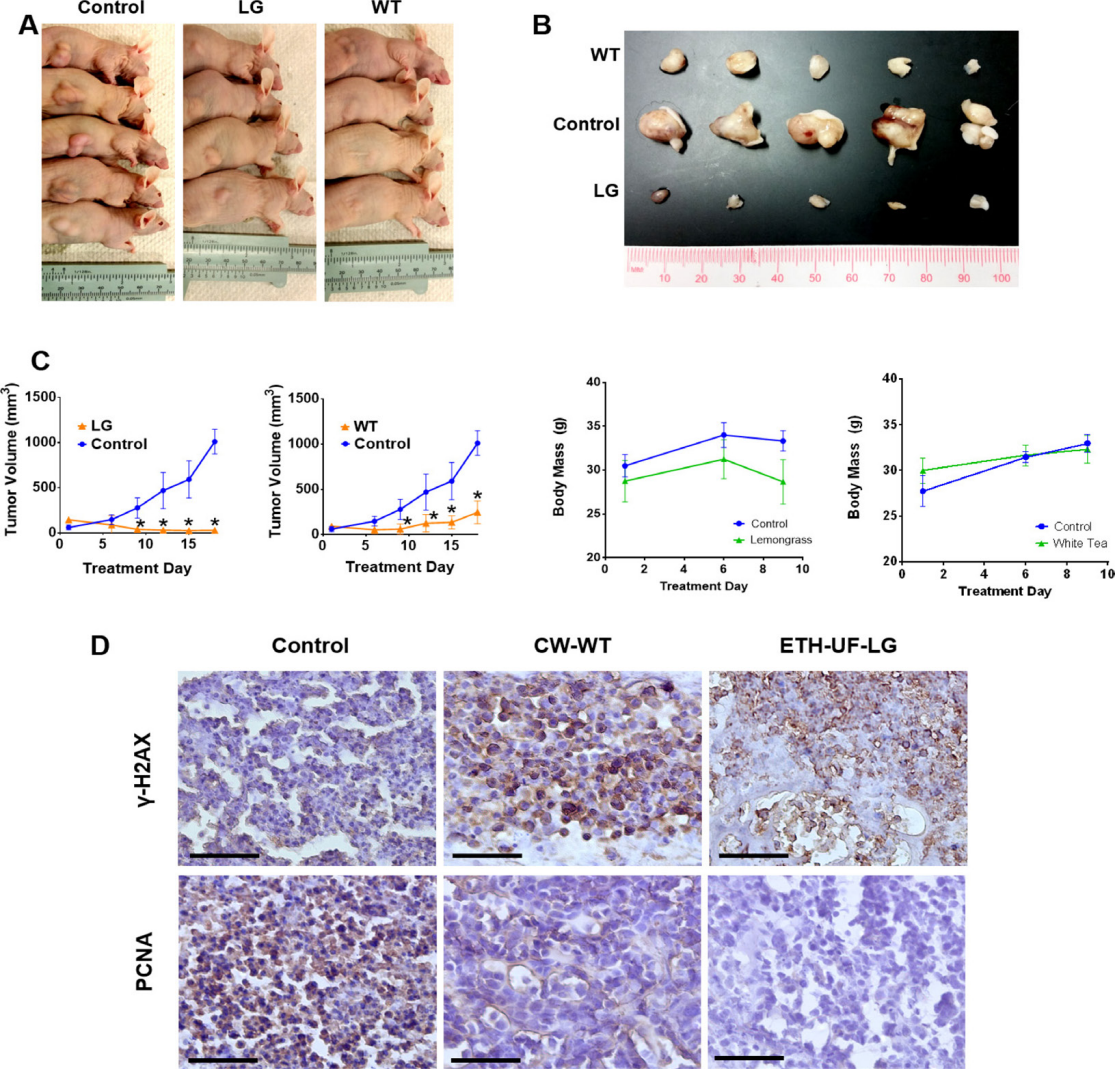
Orally administered lemongrass and white tea extract reduce tumor size in lymphoma xenograft model in immunocompromised mice Immunocompromised mice were subcutaneously injected with cancerous cells and tumors were allowed to establish. Treatments occurred every other day and the studied compound or the equivalent vehicle control administered orally for three weeks. (**A**, **B**) The tumors were photographed before and after extraction from the animals. (**C**) Tumor volume and mass were measured two times per week. (**D**) Immunohistochemistry analysis of sectioned tumor tissues from the lymphoma study. Each section was subjected to the specified antibody followed by a biotinylated secondary antibody. Detection was done using a DAB Peroxidase HRP Substrate Kit (brown) followed by Hematoxylin counterstaining (purple). Images were obtained using inverted bright field microscopy. Sectioning results are representative of three individual tumors. Scale bar is 50 microns. Statistical analysis using One-Way ANOVA. ^*^p < 0.05 vs tumor volume of the control.

Having seen effective induction of cell death by lemongrass and white tea extracts in several cell lines, we wanted to evaluate if this compound has the ability to inhibit growth of histiocytic lymphoma xenografts in mice. U-937 cells were transplanted subcutaneously in immunocompromised mice as described in the materials and methods. After palpable tumors were established, each treatment group was orally administered with either lemongrass or white tea extracts via supplemented drinking water over the course of three weeks. Both extracts were able to decrease the growth of the xenograft as determined by tumor volume relative to the vehicle control (Figure 7A–7C). Over the three-week study, there was no apparent change in body weight of mice in each group compared to the control, indicating that the animals were able to tolerate the administered treatments (Figure 7C). Furthermore, immunohistochemical staining was performed for tumor tissues treated by both lemongrass and white tea extracts. There was an increase in γ-H2AX phosphorylation, an indicator of DNA breaks, as well as a reduction in positive staining for PCNA, a marker for cellular proliferation, in both the lemongrass and the white tea extract-treated groups compared to the control group in the tumor sections (Figure 7D). Thus, these findings illustrate that lemongrass and white tea extracts are effective in reducing tumor growth *in vivo* when administered orally.

### Phytochemical analysis of ethanolic lemongrass extract

As shown in the results above, since the unfiltered, ethanolic lemongrass extract has the most effective and selective anti-cancer activity, we performed a phytochemical analysis of this particular extract. Commonly occurring compounds (34 in all) were selected for detection based on published literature searched with the key word Cymbopogon. Elimicin and lonicerin were identified based on a spectral library of over 900 phytochemicals which we have recorded in UNIFI software (Supplementary Tables 1, 2). Methyl isoeugenol was putatively identified based on monoisotopic mass observed in electrospray ionization on a Q-TOF within mass accuracy of 5 PPM and compared to published literature. Spectra of an additional 283 phenolic compounds in our in-house library of standards were matched with the spectra obtained in positive and negative modes. Commonly known phenolics were not present in the extract.

Three compounds identified in the extract (elemicin, lonicerin, and methylisoeugenol) were evaluated for their anti-cancer activity, both alone and in combination, as these were hypothesized to potentially be the active compounds contributing to the anti-cancer activity of lemongrass extract. However, our results show very poor anti-cancer activity, if at all, at very high doses (Supplementary Figure 1). Further work with other phytochemical classes will be required with purification of each compound and activity analysis.

## DISCUSSION

We have shown for the first time that lemongrass and white tea extracts exhibit different mechanisms of action for the induction of apoptosis in human lymphoma and leukemia cell lines *in vitro* and are effective in reducing tumor growth *in vivo* (Figures 2A, 5A, 7A). The induction of apoptosis by lemongrass extract was dependent on the generation of ROS while white tea extract induced apoptosis independent of oxidative stress. Both extracts were effective at inhibiting the growth of human non-Hodgkin’s lymphoma xenografts in nude mice when administered orally (Figure 7A). As they are effective in reducing the growth of subcutaneously transplanted tumors after oral administration, this result indirectly indicates that the bioactive compounds in these extracts are absorbed and stable in physiological systems.

We evaluated the efficacy of the lemongrass and white tea extracts for their activity against various lymphoma cancers. We used water and ethanolic extraction only as these two are biocompatible solvents and the extracts prepared by these procedures are considered Natural Health Products (NHPs). Of particular note, the ethanolic, unfiltered (ETH-UF) lemongrass (LG) extract had the greatest cytotoxic properties on these cancers compared to the other extraction methods (cold water, hot water and filtered, ethanolic) with EC_50_ values well below 0.08mg/mL for all four cancer cell lines while the cold and hot water extractions (CW and HW) for white tea (WT) appeared to be slightly more effective compared to their ethanolic counterparts with EC_50_ values below 0.2mg/mL (Figure 1, Table 1). Thus, these results illustrate how the extraction procedure is an important factor to consider when examining the use of whole extracts for potential health research avenues. Furthermore, these results imply that the yield of different bioactive molecules are extraction-method dependent and ultimately affect the resultant potency of each extract, a phenomenon observed previously in literature [14]. Such molecules could be specific primary (e.g. amino acids, fatty acids, sugars) or secondary (e.g. carotenoids, terpenoids, alkaloids) metabolites, which may be acquired or lost through the type of solvent used for extraction (water or ethanol) and if the extract is filtered or not.

We tested extracts from 28 different plant materials (used traditionally in various cultures) and found that these two extracts were among the few that showed significant anti-cancer activity against human lymphoma cells at reasonably low concentrations. Further characterization of the induction of cell death by ETH-UF-LG and HW-WT was performed as they were found to be the most efficacious of the four different extracts. As previous results demonstrated, both ETH-UF-LG and HW-WT were effective at inducing cell death by apoptosis in a dose-dependent manner in 3 lymphoma cell lines as indicated by the presence of Annexin V and propidium iodide staining (Figure 2). Moreover, the lowest effective doses of LG (0.05 & 0.1 mg/mL) and WT (0.1 & 0.25 mg/mL) were unable to induce cell death appreciably in a normal human fibroblast cell line (Figure 3A). LG extract was also well tolerated in peripheral nucleated blood cells (PNBCs) whereas white tea induced apoptosis in PNBCs at higher doses (Figure 3B). However, both extracts were well-tolerated by mice when administered orally over a period of three weeks.

The induction of apoptosis has been shown to be achieved by extrinsic and intrinsic mechanisms, both which converge towards the destabilization of the mitochondria. Indeed, we have demonstrated that both ETH-UF-LG and HW-WT cause mitochondrial dysfunction in a dose- and time-dependent manner as seen by monitoring the mitochondrial membrane potential dissipation (Figure 4A) and a decrease in mitochondrial oxygen consumption relative to the control caused by these natural extracts (Figure 4B). The reduced oxygen consumption reflects dysfunctional complexes of electron transport chain. Interestingly, despite both natural extracts converging towards mitochondrial collapse and the induction of apoptosis, the initial requirement of the generation of ROS for cytotoxicity is extract-specific (Figure 5). HW-WT does not appreciably induce ROS in lymphoma and leukemia cells in contrast to ETF-UF-LG. ETH-UF-LG causes ROS generation and requires the generation of oxidative stress to yield mitochondrial membrane potential collapse and the cleavage of intrinsically and extrinsically related capases-8 and -9 leading to the subsequent execution of apoptosis. Both initiation of the intrinsic and extrinsic pathways have been associated with ROS production [15] and these results offer further confirmation. Thus, it is plausible to propose that several bioactive molecules found within ETH-UF-LG increase the production of ROS and/or decrease the antioxidant capacity of the cancer cells leading to the activation of apoptosis.

As previously noted, ROS levels in cancer cells have been reported to be markedly higher compared to their normal counterparts. Through the stimulus of growth- competence genes and metabolic alterations, ROS can promote cell growth and proliferation, and may be a key factor in the abnormal rates of proliferation observed in cancer cells [16, 17]. The exact mechanism of how this occurs is not clear, but it is hypothesized that these high levels of ROS are able to activate a variety of pathways related to a cellular stress mechanism. Consequently, this may lead to the ability of cancer cells to propagate more freely within their environment [18]. It is important to note that these high levels of ROS may in fact render cancer cells more sensitive to additional ROS production and can lead to apoptotic events [18–21]. Indeed, our results have indicated that lemongrass extract treatment leads to the increased generation of ROS followed by cell death. Furthermore, the increase in the production of ROS is a critical step for the induction of apoptosis by lemongrass extract as pre-treatment with antioxidant N-acetylcysteine (NAC) blocked the induction of apoptosis (Figure 5).

However, HW-WT was still able to cause cleavage of caspase-8, and double-stranded DNA breaks as indicated by positive staining for λ-H2AX independent of ROS generation (Figures 5C, 6A), thus, illustrating how different natural extracts can yield apoptotic induction by different means (ROS vs. non-ROS mediated).

Another important finding was highlighted when examining the effects of ETH-UF-LG and HW-WT against Jurkat cells lacking a functional Fas associated death domain (FADD) protein, which is involved in the activation of the extrinsic pathway of apoptosis [22] (Figure 6B–6D). WT was effective at inducing cell death by apoptosis in these dominant-negative FADD (dnFADD) Jurkat cells at both 0.1 and 0.25 mg/mL and was unable to produce ROS. This finding supports the previous results showing that the activity of WT is independent of producing oxidative stress for its cytotoxic activity and is independent of death-inducing signalling complex (DISC). On the contrary, LG was unable to induce appreciable cell death, but was still able to induce ROS generation, in the dnFADD Jurkat cells [23, 24]. This suggests that ROS production is required before activation of proteins, such as caspase-8, for its apoptotic activity. As a whole, it is possible that the activation of caspase-8 by ROS via FADD protein could be the initial event required for the downstream apoptotic events to occur (e.g. mitochondrial collapse, activation of caspase-3), which warrants further characterization. Previous work has demonstrated multiple roles that FADD protein may have in inflammatory or pro-death pathways via cell surface receptor activation [25–27]. Therefore, ROS may be able to induce the activation of protein(s) involved in the recruitment of FADD to oligomerize, forming active DISCs.

Due to the activity of both ETH-UF-LG and HW-WT with their ability to induce cell death in various blood cancer cell lines, we wanted to explore the ability of these natural extracts to reduce xenograft tumors in nude mice (Figure 7). A major drawback of natural extract treatments is their bioavailability and stability of the bioactive compounds [28, 29]. Furthermore, these treatments cannot be administered intravenously due to the potential for immune reactions from these complex mixtures of primary and secondary metabolites. Therefore, it is important to evaluate these natural extracts for their efficacy in reducing tumor growth when administered orally. Indeed, our results demonstrated that orally administered and supplemented drinking water with ETH-UF-LG and HW-WT remarkably reduced tumor growth, decreased PCNA expression, and caused double-stranded DNA breaks in the tumors cells relative to the control. Interestingly, lemongrass extract has been shown to have inhibitory effect on carcinogen-induced colon cancer [30]. Furthermore, there was no observable decrease in the activity of the mice as well as no effect on their weight profile during the course of treatment, indicating that these natural extracts are well tolerated. This illustrates the ability of these natural extracts to be effective not only *in vitro*, but *in vivo* as well. These results also indicate that the bioactive components of these extracts are absorbed and distributed to the tumor sites to affect tumor growth.

The selectivity and efficacy of lemongrass extract’s anticancer activity incited us to perform a phytochemical analysis to identify potential active compounds. Among the reported compounds in Cybopogon spp are at least 65 essential oil compounds, with especially abundant compounds such as citral, neriol and geraniol, which were not detected in the present extract. However, most published work is on distilled essential oils while the current study was conducted with ethanol lab extracts which was subsequently dried, representing standard medicinal plant extraction methods. Under these conditions, many of the volatile compounds appear to have been removed, leaving larger molecular weight and/ or less volatile compounds. These results suggest that commonly reported compounds should not be taken for granted and that the use of untargeted or use of large reference databases are useful for extracts. Further work with other phytochemical classes are recommended.

We evaluated three compounds (elemicin, lonicerin, and methyl isoeugenol) for their anticancer activity with a lymphoma cell line as potential active compounds in lemongrass extract (Supplementary Figure 1). These compounds were ineffective in inducing apoptosis in these cells both alone and in combination, except with a combination of very high doses. This indicates the importance of multi-chemical combinations of natural extracts in inducing cell death selectively in cancer cells. The observed activity of lemongrass extract could be the result of more than three compounds acting together.

These results reveal that key compound(s) found in ETH-UF-LG are required to produce ROS-induced extrinsic apoptosis for its anti-cancer activity while HW-WT induced cell death by ROS-independent means, demonstrating that different natural extracts could lead to differential activation of cell death pathways for their anti-cancer effect. Thus, these findings open up a new opportunity for developing natural extracts as potential anti-cancer treatment alternative.

## MATERIALS AND METHODS

### Lemongrass and white tea extraction

The water and 100% ethanolic extracts of each plant were made from the stems of lemongrass and white tea leaves. The two plant products were purchased in an already pre-ground form from Premier Herbal (Toronto, Ontario, Canada) which is a wholesale supplier for various natural materials. The provided lot numbers for the lemongrass (grown in Guatemala) and white tea (grown in China) are 315872 and WT1406KIT, respectively.

For the water extracts, before extracting, water was boiled (100°C) and then allowed to cool (until ∼60°C). Each plant was then extracted with a 1:10 ratio of plant material (in grams) to distilled water (in mL) for three hours. The plant material was removed using cheesecloth, and the filtrate was centrifuged. The filtrate was then gravity-filtered using a P8 filter followed by vacuum filtration using a 0.45 μm filter. Afterwards, the filtrate was then placed in a fridge at a temperature of –80°C overnight and freeze-dried. The extracted residue left behind was weighed and reconstituted to make 100 mg/mL stock solutions.

For the ethanolic extracts, each plant was extracted with a 1:10 ratio of plant material (in grams) to anhydrous ethanol (in mL). The solution was thoroughly mixed and allowed to extract for approximately 24 hours. Following extraction, the mixture was vacuum-filtered using a 0.2 μm filter, and the filtrate underwent rotary evaporation at a temperature of approximately 38–40°C. Now, with the ethanol evaporated, the weight of the dry resin was used to make a stock solution dissolved in anhydrous ethanol. The final concentration of the extract was 100 mg/mL.

### Cell culture

The MV-4-11 Chronic myelomonocitic leukemia cell line (ATCC, Cat. No. CRL-9591, Manassas, VA, USA) was cultured with Iscove’s Modified Dulbecco’s Medium (ATCC, Cat. No. 30-2005, Manassas, VA, USA) supplemented with 10% (v/v) FBS standard (Thermo Scientific, Waltham, MA, USA) and 10 mg/mL gentamicin (Gibco BRL, VWR, Mississauga, ON, Canada).

The U-937 non-Hodgkin’s histiocytic lymphoma cell line (ATCC, Cat. No. CRL-1593.2, Manassas, VA, USA) was cultured with Iscove’s Modified Dulbecco’s Medium (ATCC, Cat. No. 30-2005, Manassas, VA, USA) supplemented with 10% (v/v) FBS standard (Thermo Scientific, Waltham, MA, USA) and 10 mg/mL gentamicin (Gibco BRL, VWR, Mississauga, ON, Canada).

The L-540 Hodgkin lymphoma (Leibniz-Institu DSMZ, Cat. No. ACC 72, Braunschweigh, Germany), was cultured with RPMI-1640 medium (Sigma-Aldrich Canada, Mississauga, ON, Canada) supplemented with 20% (v/v) fetal bovine serum (FBS) standard (Thermo Scientific, Waltham, MA, USA) and 10 mg/mL gentamicin (Gibco BRL, VWR, Mississauga, ON, Canada).

The HD-MYZ Hodgkin lymphoma (Leibniz-Institu DSMZ, Cat. No. ACC 346, Braunschweigh, Germany), was cultured with RPMI-1640 medium (Sigma-Aldrich Canada, Mississauga, ON, Canada) supplemented with 10% (v/v) fetal bovine serum (FBS) standard (Thermo Scientific, Waltham, MA, USA) and 10 mg/mL gentamicin (Gibco BRL, VWR, Mississauga, ON, Canada).

The KM-H2 Hodgkin lymphoma (Leibniz-Institu DSMZ, Cat. No. ACC 8, Braunschweigh, Germany), was cultured with RPMI-1640 medium (Sigma-Aldrich Canada, Mississauga, ON, Canada) supplemented with 10% (v/v) fetal bovine serum (FBS) standard (Thermo Scientific, Waltham, MA, USA) and 10 mg/mL gentamicin (Gibco BRL, VWR, Mississauga, ON, Canada).

The E6-1 Jurkat (acute T-cell leukemia) and Jurkat dominant negative for the Fas-Associated Death Domain (DN FADD Jurkat) cell lines (American Type Culture Collection, Cat. No. TIB-152 & CRL-2572 Manassas, VA, USA) were cultured with RPMI-1640 medium (Sigma-Aldrich Canada, Mississauga, ON, Canada) supplemented with 10% (v/v) fetal bovine serum (FBS) standard (Thermo Scientific, Waltham, MA, USA) and 10 mg/mL gentamicin (Gibco BRL, VWR, Mississauga, ON, Canada).

The normal-derived colon mucosa NCM460 cell line (INCELL Corporation, LLC., San Antonio, TX, USA) was grown in INCELL’s M3Base™ medium (INCELL Corporation, LLC., Cat. No. M300A500) supplemented with 10 % (v/v) FBS and 10 mg/mL gentamicin (Gibco BRL, VWR, Mississauga, ON, Canada).

The normal human skin fibroblasts (NHF; Coriell Institute for Medical Research, Cat. No. AG09309, Camden, NJ, USA) were grown in Dulbecco’s Modified Eagle’s Medium, High Glucose (Thermo Scientific, Waltham, MA, USA) supplemented with 15% (v/v) FBS and 10 mg/mL gentamicin (Gibco BRL, VWR, Mississauga, ON, Canada).

All cells were grown in optimal growth conditions of 37°C and 5 % CO_2_. Furthermore, all cells were passaged for less than 6 months and the authors performed no authentication of cell lines.

### Culture and isolation of peripheral blood mononuclear cells (PBMCs)

All experiments involving human subjects (healthy volunteer donating blood) were done with prior approval of Research Ethics Board of the University of Windsor (protocol # REB #04-147), with informed consent obtained from the subject. Peripheral blood mononuclear cells (PBMCs) were isolated from a healthy volunteer in BD Vacutainer CPT Tubes with Sodium Heparin^N^ (Becton, Dickinson and Company, Cat. No. 362753, Franklin Lakes, NJ, USA) at room temperature. Tubes were inverted 5 times and centrifuged for 30 minutes at room temperature at 1500–1800 × g. The PBMC layer under the plasma layer in each tube was collected, pooled together, resuspended in 50 mL of PBS, and centrifuged at room temperature at 300 × g for 15 minutes. The supernatant was aspirated without disturbing the pellet and PBMCs were suspended and cultured in RPMI-1640 medium (Sigma-Aldrich Canada, Mississauga, ON, Canada), supplemented with 10% (v/v) FBS standard (Thermo Scientific, Waltham, MA, USA) and 10 mg/mL gentamicin (Gibco BRL, VWR, Mississauga, ON, Canada) at 37°C and at 5% CO_2_. PBMCs from healthy volunteers 1 (PBMCs V1) were taken from a healthy 28-year-old male.

### WST-1 assay for cell viability

The WST-1 based colorimetric assay (Roche Applied Science, Indianapolis, IN, USA) was performed to quantify cell viability as a function of cellular metabolism. 96-well clear bottom tissue culture plates were seeded with cells. The cells were treated at the indicated concentrations and time points. The treated cells were incubated with WST-1 reagent for 4 hours at 37°C with 5% CO_2_. In actively metabolizing cells, the WST-1 reagent is cleaved to formazan by cellular enzymes. The presence of formazan was quantified via absorbance readings at 450 nm on a Wallac Victor^3^ 1420 Multilabel Counter (PerkinElmer, Woodbridge, ON, Canada). Cellular viability through measured absorbance readings expressed as percentages of the solvent control group.

### Analysis of cell death: annexin V binding assay and propidium iodide (PI)

Annexin V binding assay and propidium iodide staining were performed to respectively monitor early apoptosis and cell permeabilization, a marker of necrotic or late apoptotic cell death. Cells were washed with phosphate buffer saline (PBS) and suspended in Annexin V binding buffer (10 mM HEPES, 140 mM NaCl, 2.5 mM CaCl2, pH 7.4) with green fluorescent Annexin V AlexaFluor-488 (1:20) (Life Technologies Inc, Cat. No. A13201, Burlington, ON, Canada) and 0.01 mg/mL of red fluorescent PI (Life Technologies Inc, Cat. No. P3566, Burlington, ON, Canada) for 15 minutes at 37°C protected from light. Percentage of early (green), late apoptotic cells (green and red), and necrotic cells (red) were quantified with a Tali Image-Based Cytometer (Life Technologies Inc., Cat. No. T10796, Burlington, ON, Canada). Cells from at least 18 random fields were analyzed using both the green (ex. 458 nm; em. 525/20 nm) and red (ex. 530 nm; em. 585 nm) channels. Fluorescent micrographs were taken at 400x magnification using LAS AF6000 software with a Leica DMI6000 fluorescent microscope (Wetzlar, Germany). Cells monitored with microscopy were counterstained with Hoechst 33342 (Molecular Probes, Eugene, OR, USA) with a final concentration of 10 μM during the 15-minute incubation.

### Monitoring the mitochondria membrane potential

Tetramethylrhodamine methyl ester (TMRM) (Gibco BRL, VWR, Mississauga, ON, Canada) was used for detecting mitochondrial membrane potential (MMP), an indicator of healthy intact mitochondria, Cells monitored with microscopy were counterstained with Hoechst 33342 as previously described [5] . Images were taken with a Leica DMI6000 fluorescent microscope (Wetlar, Germany) at 400x magnification using LAS AF6000 software. JC-1 was also utilized for quantification of mitochondria membrane potential. Following treatment, JC-1 (Thermo Scientific, Cat. No. T3168, Waltham, MA, USA) at a concentration of 2 micromolar was added and allowed to incubate for 30 minutes. Following incubation, cells were washed twice in 1xPBS then analyzed using the Tali Image-Based Cytometer (Life Technologies Inc., Cat. No. T10796, Burlington, ON, Canada). Cells from at least 18 random fields were analyzed using the red (ex. 530 nm; em. 585 nm) channel.

### Oxygen consumption quantitation

Mitochondrial function was evaluated with the MitoXpress^®^ Xtra - Oxygen Consumption Assay [HS Method] (Luxcel Biosciences Ltd., Cat. No. MX-200, Cork, Ireland). 1 000 000 cells/well were seeded in a 96-well black clear bottom tissue culture plate and incubated for an hour at 37°C and 5% CO_2_. On a heat pack, 10 μL of MitoXpress^®^ reagent was added to each well excluding the blanks, cells were treated, the plate was shaken with a plate shaker, and 2 drops of pre-warmed high sensitivity mineral oil was added to each well to seal off the air supply. Bottom read fluorescence measurements were taken at Ex. 380 nm and Em. 650, every 2 minutes for 2 hours at 37°C using a SpectraMax Gemini XS multi-well plate reader (Molecular Devices, Sunnyvale, CA, USA). Increases in fluorescence are indicative of oxygen consumption. Oxygen consumption rates were determined by calculating the slope of the linear regions of the oxygen consumption curves using GraphPad Prism 6 software.

### Quantitation of reactive oxygen species (ROS)

Whole cell ROS generation was monitored with the small molecule 2′, 7′-dicholorofluorescin diacetate (H_2_DCFDA). H_2_DCFDA enters the cell and is deacetylated by esterases and oxidized by ROS to the highly fluorescent 2′, 7′-dicholorofluorescein (DCF) (excitation 495 nm; emission 529 nm). Cells were pretreated with 20 μM H_2_DCFDA (Sigma-Aldrich Canada, Cat. No. D6883, Mississauga, ON, Canada) for 30 minutes at 37°C protected from light at 5% CO_2_. Cells were treated for the indicated durations, centrifuged at 600 × g for 5 minutes and suspended in PBS. Percentage of DCF positive cells was quantified using the Tali Image-Based Cytometer (Life Technologies Inc., Cat. No. T10796, Burlington, ON, Canada) using 12 random fields per group with the green channel (excitation 458 nm; emission 525/20 nm).

### *In vivo* xenograft model and extract administration

All experiments involving animals (mice) and animal protocols were approved by the University of Windsor Animal Care Committee (AUPP # 14–15) in accordance with the Canadian Animal Care Committee. Immunocompromised CD-1 nu/nu male mice (Charles River Laboratories, Cat. No. 086, Sherbrooke, QC, Canada) were housed in laboratory conditions of a 12-hour light/dark cycle. Mice were injected using 23-gauge needles with 1 mL syringes with equal amounts of cell solution (containing 2 × 10^6^ U-937 cells) and Corning Matrigel Basement Membrane Matrix (VWR International, Cat. No. 47743-715, Missisauga, ON, Canada) to a final volume of 200 microliters. Once tumors were established, the animals were randomized into three groups of 4 mice each including a control, treatment with LG, and treatment with WT. The control group was housed with a bottle of regular drinking water while the treatment groups were given water which contained the respective treatments. The water was changed regularly twice per week and the amount drank was measured. Each mice in the treatment groups was calculated to have consumed, on average, 80 mg/kg/day of the respective treatment. The volumes of the tumours were measured according to their length, width, and height twice per week and calculated using the ellipsoid formula п/6 × length × width × height. Changes in body mass were measured with a scale to assess for potential weight loss and determine whether treatments were well tolerated.

### Cell lysis and western blot analysis

Preparation of cell lysates and Western blot analysis were performed as previously described [5]. In brief, protein samples were run using SDS-PAGE and transferred to PVDF membranes. Membranes were blocked for one hour with either 5% skim milk or bovine serum albumin (BSA) at room temperature. Following blocking, the membranes were incubated with one of the following primary antibodies overnight at 4°C: anti-caspase-8 antibody (1:1000) raised in mouse (Cell Signalling, Cat. No. 9746 S, Danvers, MA, USA), anti-caspase-9 antibody (1:1000) raised in rabbit (Cell Signalling, Cat. No. 9502, Danvers, MA, USA), anti-caspase-3 antibody (1:2000) (Novus Biologicals, Cat. No. NB100-56709V2, Littleton, CO, USA), anti-β-actin antibody (1:1000) (Santa Cruz Biotechnology, Inc., Cat. No. sc-81178, Paso Robles, CA, USA), anti-p-Histone H2A.X (Ser 139) (γ-H2AX) antibody (Santa Cruz Biotechnology, Inc., Cat. No. sc-101696, Paso Robles, CA, USA), and anti-vinculin antibody (1:2000) raised in rabbit (Cell Signalling, Cat. No. 13901, Danvers, MA, USA)

### Cryosectioning and immunohistochemistry

Following the *in vivo* study, U937 tumors were harvested and placed a 10% formaldehyde solution. Three days prior to sectioning, they were transferred to 30% sucrose (w/v). Tumors were then sectioned at 20 µm and subjected to immunohistochemistry using either gamma H2AX (p Ser139) antibody (1:500) raised in rabbit (Novus Biologicals, Cat. No. NB100-384, Littleton, CO, USA), active/cleaved Caspase 8 antibody (1:500) raised in rabbit (Novus Biologicals, Cat. No. NB100-56116, Littleton, CO, USA), or anti-PCNA [pc10] antibody (1:400) raised in mouse (Abcam, Cat. No. ab29, Cambridge, MA, USA). Prior to overnight incubation with these primary antibodies at 4°C, the sections were incubated in 0.33% H_2_O_2_ for 2 minutes in order to block endogenous peroxidases, DAKO universal blocking solution (purchased from Diagnostics Canada Inc., Mississauga) for 30 minutes, and in normal goat serum (for primary antibodies raised in rabbit) or normal horse serum (for primary antibodies raised in mouse) for 30 minutes at room temperature (prepared as per instructions on anti-rabbit Vecstatin ABC Kit [Cat. No. PK-6101] or anti-mouse Vecstatin ABC Kit [Cat. No. PK-6102], Vector Laboratories) in order to block the binding of non-specific goat or horse IgG. The sections were washed in Tris buffered saline (TBS) for 5 minutes in between each blocking step to remove any excess blocking reagents. Following the overnight incubation, the sections were washed in TBS twice and were incubated in biotinylated anti-rabbit IgG (Vector Laboratories, anti-rabbit Vecstatin ABC Kit [Cat. No. PK-6101]) or biotinylated anti-mouse IgG (Vector Laboratories, anti-rabbit Vecstatin ABC Kit [Cat. No. PK-6102]) for 75 minutes at room temperature. The sections were then washed twice with TBS for 5 minutes and were incubated in avidin biotin complex (ABC reagent) for 45 minutes at room temperature. Following two additional TBS washes, the sections were subjected to the peroxidase substrate 3, 3′ diaminobenzidine (DAB) prepared as per the instructions in the kit (Vector Laboratories, DAB Peroxidase (HRP) Substrate Kit [Cat. No. SK-4100]). Sections were then counterstained with Hematoxylin Solution, Gill No. 1 (Sigma-Aldrich Canada, Cat. No. GHS116, Mississauga, ON, Canada) and washed twice in tap water for 5 minutes. The sections were then washed in Scott’s Bluing solution 5 minutes, followed by a 5-minute wash in TBS. The sections were then dehydrated in anhydrous ethanol and xylene and were cover-slipped using Permount for visualization under a microscope.

### Statistical analysis

Statistics from this study were performed with GraphPad Prism 6 statistical software. A *p*-value below 0.05 was considered significant. For the experiments with single variable measurements, which include quantification of MMP, and whole cell ROS, a One-Way ANOVA (nonparametric) was conducted and each sample’s mean was compared to the mean of the negative control (DMSO vehcile) unless otherwise specified. For experiments that contained multi-variables (e.g. multiple group comparisions), such as the quantification of live and dead cells, Two-Way ANOVA (nonparametric) was used and each sample’s mean was compared to the mean of the negative control (DMSO vehcile) unless otherwise specified.

### Sample preparation for phytochemical analysis

The lemongrass extract LG072416 (10 mg) was dissolved in 1 mL dimethyl sulfoxide (DMSO), and diluted with 50% methanol + 50% water + 0.1% formic acid to yield a final concentration of 10 µ/mL. The diluted extract was sonicated for 5 min and filtered though 0.2 µ syringe filter.

### Analytical method

Analysis was performed with an ultraperformance Liquid chromatograph connected with quadrupole time of flight mass spectrometer UPLC-QTOF (Waters Acquity Xevo G2 QTOF, Waters Corp. Separation was achieved with a reverse phase column: Guard Filter, PN 289002378 + Acquity BEH C18 1.7um 2.1 × 50 mm. The mobile phase solvents were Fisher Optima LC-MS grade (Fisher Scientific, Ottawa ON), flow rate: 0.8 mL/min. The column temperature was 50°C, and autosampler: 4°C. Sample injection (PLUNO was 5uL, with a post injection needle wash: 200 uL (50% acetonitrile + 50% water) + weak wash 600 uL (10% acetonitrile + 90% water). The mobile phase: A1: water+0.1% formic acid, B1: Acetonitrile + 0.1% formic acid.

The QTOF was used with the following conditions: sourcee 400°C, cone gas (N2) flow 50 L/h, desolvation gas (N2) 1200 L/h, Cone voltage 35V, Scan time 0.08 sec. Calibration, 100–1500 Da. MassLynx software, MSe ESI+ mode. Enkephalin m/z556.2615 was used as a reference (ESI pos), source temperature 120°C. Scans were 100–1500 Da, F1: CE, 6V, F2: CER 20–50V (both negative and positive ionization modes). A 28 min step gradient from A1:B1 = 99%:1% to 0%:100% was used.

Compounds were identified definitively based on in house spectral library or tentatively based on monoisotipic mass observed in electrospray ionization on a QTOF within mass accuracy of 5 PPM.

## SUPPLEMENTARY MATERIALS FIGURE AND TABLES





## Abbreviations

ROS: Reactive Oxygen Species
ETC: Electron transport chain
CW: Cold water
HW: Hot water
ETH-F: Ethanolic-filtered
ETH-UF: Ethanolic-unfiiltered
WST-1: water-soluble tetrazolium-1
AMA: Antimycin A
NAC: N-acetyl cysteine
FADD: Fas-associated death domain
dnFADD: double-negative Fas-associated death domain
PCNA: Proliferating cell nuclear antigen
NHP: Natural health product
LG: Lemongrass
WT: White tea
DISC: death-inducing
DMSO: Dimethyl sulfoxide
