# Supplementary material for: Cymbopogon citratus and Camellia sinensis extracts selectively induce apoptosis in cancer cells and reduce growth of lymphoma xenografts in vivo
Source: Oncotarget. 2017 Nov 18;8(67):110756–73. doi: 10.18632/oncotarget.22502 (PMC5762282; doi:10.18632/oncotarget.22502)
Supplement: Supplementary file 1 [file oncotarget-08-110756-s001.pdf]

Cymbopogon citratus and *Camellia sinensis* extracts selectively induce apoptosis in cancer cells and reduce growth of lymphoma xenografts *in vivo*

SUPPLEMENTARY MATERIALS

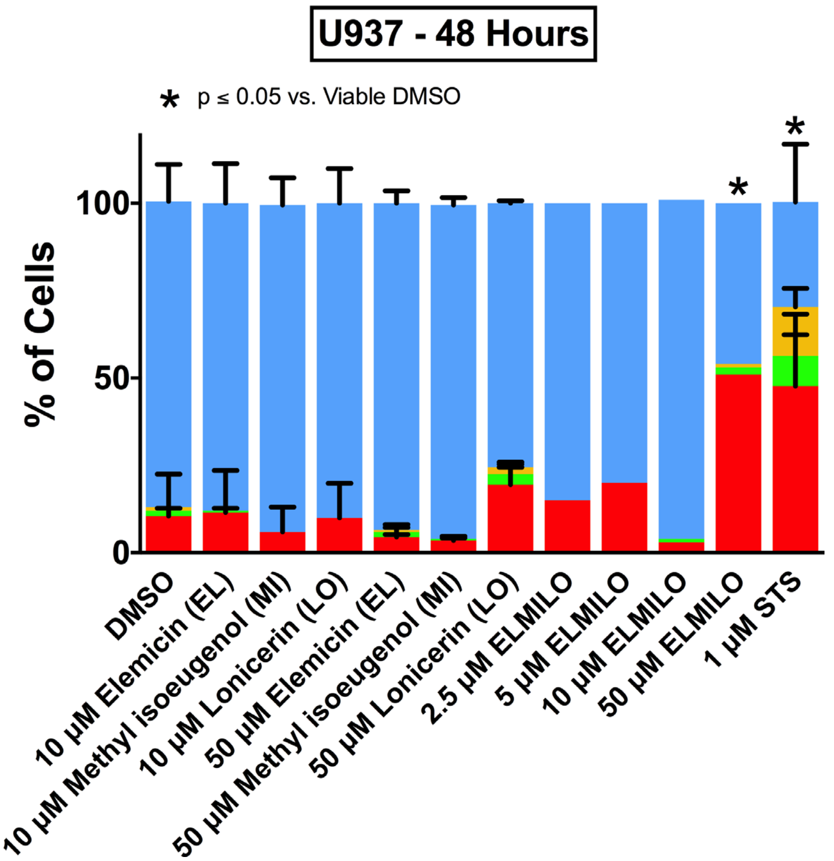

Supplementary Figure 1: Cytotoxic activity of three compounds identified in lemongrass extract individually and in combination on a lymphoma cell line.

Supplementary Table 1: List of compounds identified in lemongrass extract. See Supplementary_ Table_1

Supplementary Table 2: Three unique compounds identified in lemongrass extract

Compound	Molecular formula	Monoisotopic Mass	Calculated [M+H]+	Observed [M+H]+	Calculated [M-H]-	Retention time (min)	Comments
Elemicin	C12H16O3	208.1099	209.1178 (1+)	209.1150	207.1021 (1–)	3.00	Reported in Cymbopogon procerus
Lonicerin	C27H30O15	594.1585	595.1663 (1+)		593.1506 (1–)	3.54	Cymbopogon citratus
Methylisoeugenol	C11H14O2	178.0994	179.1072 (1+)	179.1050	177.0916 (1–)	2.43	Edited by Jiangsu New Medicinal College, Chinese Medicine Dictionary, Shanghai Science and technology Press, Shanghai, (1979)<br><br>Sun, *et al*., Brief Handbook of Natural Active Compounds, Medicinal Science and Technology Press of China, Beijing, (1998)
