# Supplementary material for: Cymbopogon citratus and Camellia sinensis extracts selectively induce apoptosis in cancer cells and reduce growth of lymphoma xenografts in vivo
Source: Oncotarget. 2017 Nov 18;8(67):110756–73. doi: 10.18632/oncotarget.22502 (PMC5762282; doi:10.18632/oncotarget.22502)
Supplement: Supplementary file 2 [file oncotarget-08-110756-s002.docx]

| **Compound** | **Molecular formula** | **Monoisotopic Mass** | **Calculated [M+H]+** | **Observed [M+H]+** | **Calculated [M-H]-** | **Retention time (min)** | **Reported in** |
| --- | --- | --- | --- | --- | --- | --- | --- |
| Dipentene | C10H16 | 136.1252 | 137.1330 (1+) |  | 135.1174 (1-) |  | Cymbopogon spp. |
| (+/-)-beta-Phellandrene | C10H16 | 136.1252 | 137.1330 (1+) |  | 135.1174 (1-) |  | Cymbopogon flexuosus |
| Piperitenone | C10H14O | 150.1045 | 151.1123 (1+) |  | 149.0966 (1-) |  | Cymbopogon martinii |
| (E)-Citral | C10H16O | 152.1201 | 153.1279 (1+) |  | 151.1123 (1-) |  | Cymbopogon citratus |
| (E)-Citral | C10H16O | 152.1201 | 153.1279 (1+) |  | 151.1123 (1-) |  | Cymbopogon distans |
| (E)-Citral | C10H16O | 152.1201 | 153.1279 (1+) |  | 151.1123 (1-) |  | Cymbopogon flexuosus |
| (Z)-Citral | C10H16O | 152.1201 | 153.1279 (1+) |  | 151.1123 (1-) |  | Cymbopogon citratus |
| (Z)-Citral | C10H16O | 152.1201 | 153.1279 (1+) |  | 151.1123 (1-) |  | Cymbopogon flexuosus |
| (+)-Piperitone | C10H16O | 152.1201 | 153.1279 (1+) |  | 151.1123 (1-) |  | Cymbopogon senarensis |
| (+)-cis-p-Mentha-1(7),8-dien-2-ol | C10H16O | 152.1201 | 153.1279 (1+) |  | 151.1123 (1-) |  | Cymbopogon densiflorus |
| (+)-trans-p-Mentha-1(7),8-dien-2-ol | C10H16O | 152.1201 | 153.1279 (1+) |  | 151.1123 (1-) |  | Cymbopogon densiflorus |
| trans-p-Mentha-1(7),8-dien-3-ol | C10H16O | 152.1201 | 153.1279 (1+) |  | 151.1123 (1-) |  | Cymbopogon spp. |
| p-Mentha-1,8-dien-6-ol | C10H16O | 152.1201 | 153.1279 (1+) |  | 151.1123 (1-) |  | Cymbopogon martinii |
| (R)-(+)-Perillyl alcohol | C10H16O | 152.1201 | 153.1279 (1+) |  | 151.1123 (1-) |  | Cymbopogon polyneuros |
| (+)-trans-p-Mentha-2,8-dien-1-ol | C10H16O | 152.1201 | 153.1279 (1+) |  | 151.1123 (1-) |  | Cymbopogon martinii |
| (+)-cis-p-Mentha-2,8-dien-1-ol | C10H16O | 152.1201 | 153.1279 (1+) |  | 151.1123 (1-) |  | Cymbopogon martinii |
| (-)-Carvotanacetone | C10H16O | 152.1201 | 153.1279 (1+) |  | 151.1123 (1-) |  | Cymbopogon nardus |
| (1S,4R)-(+)-Isodihydrocarvone | C10H16O | 152.1201 | 153.1279 (1+) |  | 151.1123 (1-) |  | Cymbopogon martinii |
| Citral | C10H16O | 152.1201 | 153.1279 (1+) |  | 151.1123 (1-) |  | Cymbopogon citratus |
| Citral | C10H16O | 152.1201 | 153.1279 (1+) |  | 151.1123 (1-) |  | Cymbopogon flexuosus |
| Geraniol | C10H18O | 154.1358 | 155.1436 (1+) |  | 153.1279 (1-) |  | Cymbopogon distans |
| Geraniol | C10H18O | 154.1358 | 155.1436 (1+) |  | 153.1279 (1-) |  | Cymbopogon martinii |
| (R)-Citronellal | C10H18O | 154.1358 | 155.1436 (1+) |  | 153.1279 (1-) |  | Cymbopogon nardus |
| (-)-Isoborneol | C10H18O | 154.1358 | 155.1436 (1+) |  | 153.1279 (1-) |  | Cymbopogon goeringii |
| Citronellal | C10H18O | 154.1358 | 155.1436 (1+) |  | 153.1279 (1-) |  | Cymbopogon citratus |
| Citronellal | C10H18O | 154.1358 | 155.1436 (1+) |  | 153.1279 (1-) |  | Cymbopogon densiflorus |
| Citronellal | C10H18O | 154.1358 | 155.1436 (1+) |  | 153.1279 (1-) |  | Cymbopogon nardus |
| Citronellal | C10H18O | 154.1358 | 155.1436 (1+) |  | 153.1279 (1-) |  | Cymbopogon winterianus |
| (+)-R-Citronellol | C10H20O | 156.1514 | 157.1592 (1+) |  | 155.1436 (1-) |  | Cymbopogon distans |
| Diosphenol | C10H16O2 | 168.1150 | 169.1229 (1+) |  | 167.1072 (1-) |  | Cymbopogon densiflorus |
| **Methylisoeugenol** | **C11H14O2** | 178.0994 | **179.1072 (1+)** | **179.1050** | 177.0916 (1-) | **2.43** | Cymbopogon goeringii |
| Acetic acid geraniol ester | C12H20O2 | 196.1463 | 197.1542 (1+) |  | 195.1385 (1-) |  | Cymbopogon martinii |
| gamma1-Cadinene | C15H24 | 204.1878 | 205.1956 (1+) |  | 203.1800 (1-) |  | Cymbopogon nardus |
| **Elemicin** | **C12H16O3** | 208.1099 | **209.1178 (1+)** | **209.1150** | 207.1021 (1-) | **3.00** | Cymbopogon procerus |
| Isointermedeol | C15H26O | 222.1984 | 223.2062 (1+) |  | 221.1905 (1-) |  | Cymbopogon flexuosus |
| beta-Caryophyllene alcohol | C15H26O | 222.1984 | 223.2062 (1+) |  | 221.1905 (1-) |  | Cymbopogon martinii |
| (-)-Eudesm-7(11)-en-4alpha-ol | C15H26O | 222.1984 | 223.2062 (1+) |  | 221.1905 (1-) |  | Cymbopogon flexuosus |
| Cryptomeridiol | C15H28O2 | 240.2089 | 241.2168 (1+) |  | 239.2011 (1-) |  | Cymbopogon proximus |
| (-)-10-epi-5beta,11-Dihydroxyeudesmane | C15H28O2 | 240.2089 | 241.2168 (1+) |  | 239.2011 (1-) |  | Cymbopogon distans |
| Cymbodiacetal | C20H30O4 | 334.2144 | 335.2222 (1+) |  | 333.2066 (1-) |  | Cymbopogon martinii |
| (-)-beta-Sitosterol | C29H50O | 414.3862 | 415.3940 (1+) |  | 413.3783 (1-) |  | Cymbopogon flexuosus |
| **Lonicerin** | **C27H30O15** | **594.1585** | **595.1663 (1+)** |  | **593.1506 (1-)** | **3.54** | Cymbopogon citratus |
